# Coronary Intravascular Imaging: A Comprehensive Review of Techniques, Applications, and Future Directions

**DOI:** 10.3390/medicina61112019

**Published:** 2025-11-12

**Authors:** Giustina Iuvara, Marco Franzino, Gabriele Carciotto, Tommaso De Ferrari, Stefania Lo Giudice, Francesco Pallante, Federico Giannino, Manuela Ajello, Sofia Tomasi, Luigi Sciortino, Gabriele Monciino, Walter Licandri, Rodolfo Caminiti, Vittorio Virga, Francesco Costa, Antonio Micari, Giampiero Vizzari

**Affiliations:** 1 Department of Clinical and Experimental Medicine, University of Messina, 98124 Messina, Italy; giustina.iuvara@gmail.com (G.I.); gcarciotto97@gmail.com (G.C.); stefania.logiudice1996@gmail.com (S.L.G.); francescopallante98@gmail.com (F.P.); federicogianninoa@gmail.com (F.G.); manuela.ajello@outlook.it (M.A.); sofiam.tomasi@gmail.com (S.T.); luigi.sciortino98@gmail.com (L.S.); gabrymonciino@live.it (G.M.); w.licandri@gmail.com (W.L.); 2 Department of Cardiology, Division of Heart and Lungs, Utrecht University Medical Center, Utrecht University, 3584 CX Utrecht, The Netherlands; 3 Cardiology Division, Sant’Andrea Hospital, 13100 Vercelli, Italy; marco.franzino@outlook.it; 4 Interventional Cardiology Unit, University Hospital Policlinico G. Martino, 98124 Messina, Italy; vittorio.virga@polime.it (V.V.); micariantonio@gmail.com (A.M.); 5 Interventional Cardiology Unit, IRCCS San Raffaele Scientific Institute, 20132 Milan, Italy; tommaso.deferrari@outlook.it; 6 IRCCS Ospedale Galeazzi Sant’Ambrogio, 20157 Milan, Italy; rudycaminiti@gmail.com; 7 Department of Biomedical and Dental Sciences, Morphological and Functional Images, University of Messina, 98122 Messina, Italy; dottfrancescocosta@gmail.com; 8 Departamento de Medicina UMA, Área del Corazón, Hospital Universitario Virgen de la Victoria, CIBERCV, IBIMA Plataforma BIONAND, 29010 Malaga, Spain

**Keywords:** intracoronary imaging, intravascular ultrasound, IVUS, optical coherence tomography, OCT, vulnerable plaque, AI integrated software analysis

## Abstract

Intravascular imaging has revolutionized the assessment and management of coronary artery disease, providing unparalleled insights into plaque morphology, lesion severity, and percutaneous coronary intervention (PCI) optimization. This comprehensive review explores the current landscape of intravascular imaging, detailing the principles and clinical utility of intravascular ultrasound (IVUS) and optical coherence tomography (OCT). We discuss the role of these technologies in various clinical scenarios, ranging from stable coronary artery disease to acute coronary syndromes, emphasizing their ability to refine diagnostic accuracy and therapeutic decision-making. A key focus is placed on their application in identifying vulnerable plaques, a critical step in preventing adverse cardiovascular events. Furthermore, we highlight the role of intravascular imaging in guiding PCI, improving stent deployment, and reducing procedural complications. Finally, we explore emerging imaging modalities and technological advancements poised to further enhance coronary assessment, including hybrid imaging techniques. In addition to established modalities, this review examines emerging imaging technologies and the growing integration of artificial intelligence (AI) and hybrid imaging systems, which hold promise for automated plaque characterization, improved reproducibility, and enhanced decision support during PCI. By summarizing the latest evidence and future directions, this review aims to provide a comprehensive reference for clinicians and researchers seeking to optimize the use of intravascular imaging in contemporary cardiovascular practice.

## 1. Introduction

Coronary angiography represents the standard method for the diagnosis of coronary artery disease and for leading percutaneous coronary intervention (PCI) [1]. However, conventional angiography provides a coronary luminography, offering limited information about the entire vessel wall and consequently about the characterization of plaque, its distribution, and clues of suboptimal stent deployment.

Intravascular imaging provides detailed cross-sectional images of the vessel wall, defining morphology and plaque distribution before PCI, guiding the procedure for stent sizing, and helping the optimization after stent implantation, revealing predictors of outcomes such as underexpansion, malapposition, and dissection [2]. By enabling precise lesion assessment and tailored stent deployment, imaging-guided PCI has emerged as a key determinant of procedural success and long-term clinical outcomes. Its ability to minimize complications such as restenosis and stent thrombosis underscores its pivotal role in modern interventional cardiology.

Intravascular ultrasound (IVUS) and optical coherence tomography (OCT) are the most commonly used methods in clinical practice. Near-infrared spectroscopy (NIRS) imaging, allowing for the quantification of the lipid component coupled with plaque anatomy, is rarely used in clinical practice.

It is now well established that imaging-guided PCI is associated with decreased incidence of stent thrombosis and MACE, as evidenced by several published trials and meta-analysis [3,4,5,6]. In the wake of these results, the latest American Heart Association/American College of Cardiology (2025) [7] and European Society of Cardiology (2024) [8] guidelines for coronary revascularization have upgraded the indication of intravascular imaging guidance from class IIa to class I for patients undergoing stent implantation in the left main artery or in complex lesions.

Nevertheless, its use remains constrained in clinical practice [9]. Possible explanations could be the local availability and associated costs of expertise in acquiring and interpreting intravascular images. Further big limitation is represented by the extra time required for imaging.

Further evidence to support the use of intravascular imaging is expected from ongoing randomized trials comparing its long-term outcomes with angiography guidance.

Among new perspectives, cardiovascular imaging research has turned its attention on the biochemical characterization of coronary atherosclerosis and its morphologic features, with the aim of estimating the biologic behavior of the plaque and predicting future events.

A comprehensive literature search was conducted in the MEDLINE/PubMed database using combinations of the terms “percutaneous coronary intervention”, “intravascular ultrasound”, “optical coherence tomography”, “near-infrared spectroscopy”, “intravascular imaging”, and “intracoronary imaging”. The search included articles published up to October 2025 and was limited to studies on humans, encompassing randomized clinical trials, observational studies, and review articles. Additional references were identified through manual screening of the bibliographies of relevant papers.

## 2. Intravascular Imaging Technology

### 2.1. IVUS Technology

IVUS technology is based on ultrasound waves generated by piezo-electric crystals and reflected to the probe by surrounding tissue structures in order to produce monochrome images in grayscale. IVUS catheters can be broadly divided into two categories: mechanical rotational systems (annular-array) operating at a typical frequency of 40–45 MHz (e.g., OptiCross™, Boston Scientific, Marlborough, MA, USA) and phased-array systems equipped with multiple fixed transducers, usually operating at 20 MHz (e.g., Eagle Eye™, Philips Volcano, San Diego, CA, USA). The fundamental distinction between these two technologies lies in their image acquisition mechanism. Mechanical IVUS generates cross-sectional vascular images through 360° rotation at >1800 rpm, using a single rotating element; however, the mechanical motion within the vessel can produce image distortion and non-uniform rotational artifacts. In contrast, solid-state IVUS—also referred to as multi-element phased-array IVUS—constructs images through the electronic activation of fixed elements, offering improved lateral resolution due to a narrower beam profile and eliminating rotational distortion. Its limitation, however, is a lower center frequency (20 MHz), which yields inferior axial resolution (<170 μm) [10].

In commonly used systems, the axial and lateral resolutions are approximately 80–100 μm and 200–250 μm, respectively, with a tissue penetration depth of ~6–12 mm (Table 1). To overcome the limited plaque characterization capability of grayscale IVUS, several radiofrequency (RF) post-processing modalities were developed to enhance tissue characterization [11], including virtual histology IVUS (VH-IVUS), integrated backscatter IVUS (IB-IVUS), iMAP-IVUS (Boston Scientific, Marlborough, MA, USA), and automated differential echogenicity (ADE) [12]. More recently, high-definition IVUS (HD-IVUS) with a 60 MHz transducer has been introduced, providing superior axial resolution (<40 μm) [13].

### 2.2. OCT Technology

**Figure 1:**
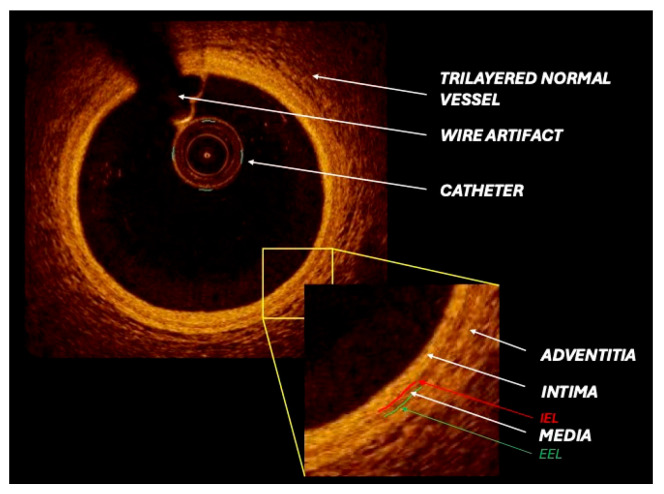
Optical coherence tomography (OCT) cross-sectional image depicts a healthy coronary artery with normal vessel wall architecture and clear visualization of all identifiable layers and components. IEL: Internal elastic lamina; EEL: external elastic lamina.

Optical coherence tomography employs a fiber-optic catheter that delivers near-infrared light within the 1250–1350 nm wavelength range, generating images with an axial resolution of approximately 10–15 μm. The imaging penetration depth is typically 1.0–2.0 mm depending on tissue composition, while the maximal scanning diameter reaches up to 10 mm. Image acquisition is performed during contrast injection (3.0–3.5 mL/s for 3–4 s) through the guiding catheter. Owing to its high frame rate (158–180 frames/s) and rapid pullback speed (36–40 mm/s), OCT enables the visualization of long coronary segments (54–75 mm) (Table 1). Image reconstruction can be displayed in cross-sectional, longitudinal, or three-dimensional formats based on operator preference (Figure 1). At present, two OCT platforms are commercially available: the frequency-domain OCT system (ILUMIEN™ OPTIS™, Abbott Vascular Inc., St. Paul, MN, USA) and the optical frequency-domain imaging system (LUNAWAVE™, Terumo Corporation, Tokyo, Japan) [14,15].

## 3. Intravascular Imaging Application in Different Clinical Scenarios

### 3.1. Chronic Coronary Syndrome

The advent of intravascular imaging has significantly enhanced the understanding and management of chronic coronary syndromes (CCSs) by allowing for the detailed assessment of atherosclerotic plaque morphology and lesion severity beyond the limitations of conventional coronary angiography.

It can easily allow us to highlight the different layers of the vessels’ walls, which appear differently based on the technique used, and to characterize the different components of the plaque. The tunica intima can be easily recognized from the lumen by its different acoustic impedance (rich signal). The tunica media is a low-signal layer placed between internal and external elastic lamina (EEL), the outer recognizable layer with IVUS imaging, since borders of adventitial and periadventitial tissue are not discernible. Adventitia is the outer signal-rich layer and consists of the adventitial and peri-adventitial tissues.

The ability to characterize plaque morphology is critical for risk stratification and therapeutic decision-making. Plaques are commonly classified into lipid-rich, fibrotic, or calcified phenotypes, each with distinct implications for plaque stability, progression, and response to intervention.

**Figure 2:**
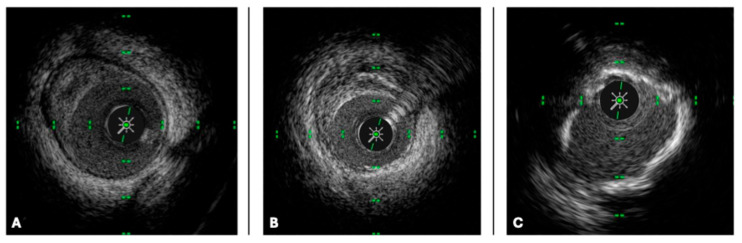
Intravascular ultrasound (IVUS) cross-sectional images of coronary arteries showing three different types of atherosclerotic plaque: (**A**) lipid plaque, (**B**) fibrotic plaque, (**C**) calcified plaque (see text).

**Figure 3:**
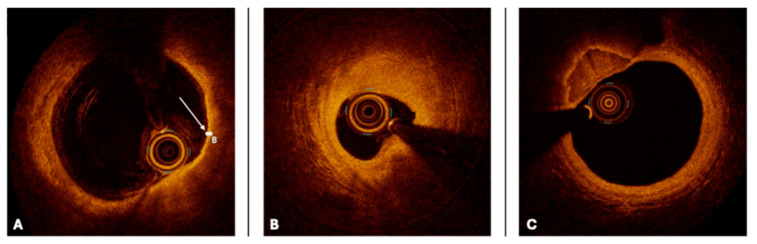
Optical coherence tomography (OCT) cross-sectional images of coronary arteries showing three different types of atherosclerotic plaque: (**A**) lipid plaque (the arrow indicates thin fibrous cap), (**B**) fibrotic plaque, (**C**) calcified plaque (see text).

Lipid-rich plaques (LRPs) are highly associated with plaque vulnerability and rupture, serving as the substrate for acute coronary syndromes (ACS). On IVUS, LRPs typically exhibit low-echoic regions with a hypoechoic core and may demonstrate positive remodeling, and as assessed by the PROSPECT study [16], non-flow-limiting lipid-rich plaques are responsible for a significant proportion of future coronary events (Figure 2). On OCT, they appear as low-signal intensity regions with diffuse signal attenuation due to high lipid content (Figure 3). A fibrous cap thickness (FCT) < 65 µm, as measured by OCT, is a hallmark of thin-cap fibroatheroma (TCFA), a well-established predictor of plaque rupture and subsequent myocardial infarction [17,18].

Fibrotic plaques represent stable atherosclerotic lesions with a lower propensity for rupture. They appear as homogeneous, high-echoic structures on IVUS and as bright, uniform regions with well-defined borders on OCT. These plaques are commonly found in patients with CCS and rarely require intervention unless associated with significant luminal narrowing, and functionally non-significant fibrotic plaques may not derive substantial benefit from PCI [19].

Calcified plaques pose a significant challenge in PCI, often leading to suboptimal stent expansion and increased procedural complexity. On IVUS, they appear as bright, highly reflective areas with acoustic shadowing, while on OCT, they are seen as well-demarcated, high-signal intensity regions with signal dropout behind the calcified zone. The degree of calcification influences procedural strategy, as severely calcified lesions may require lesion preparation with rotational atherectomy, orbital atherectomy, or intravascular lithotripsy to facilitate optimal stent deployment [20,21]. The ILUMIEN III study [22] demonstrated that OCT-guided stent implantation resulted in superior stent expansion in calcified lesions compared to angiography-guided PCI.

A validated OCT-derived calcium scoring system allocates one point for calcium thickness exceeding 0.5 mm, one point for a longitudinal calcium extension greater than 5 mm, and two points for a calcium arc > 180°. Notably, severe stent underexpansion occurred when all components were present, yielding a total score of 4 points [23]. Beyond nodular calcifications, OCT also allows for differentiation between superficial and deep non-nodular calcium deposits, with superficial calcium being more likely to impair stent expansion and vessel compliance, while deep calcium primarily affects overall plaque rigidity. This distinction provides valuable insights for procedural planning and calcium modification strategies.

A newly validated, refined OCT-based calcium scoring system has been introduced to more accurately predict stent underexpansion in heavily calcified lesions, particularly those exhibiting a calcium arc greater than 270° [24]. In this score, one point is assigned to each of the following morphologic features: minimum calcium thickness > 0.3 mm; calcium arc of 360°; calcium segment length (with arc > 270°) > 3 mm.

**Figure 4:**
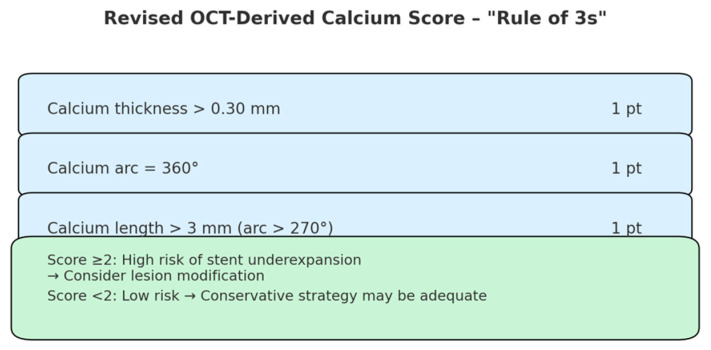
Revised OCT-derived calcium score (“Rule of 3s”) for predicting stent underexpansion in calcified lesions (adapted from “A Revised Optical Coherence Tomography–Derived Calcium Score to Predict Stent Underexpansion in Severely Calcified Lesion” [24]).

This three-point scoring system, also known as the “rule of 3s” (Figure 4), demonstrated a stepwise inverse relationship with stent expansion. Stent underexpansion (defined as <70% expansion) occurred significantly more frequently in lesions with a calcium score ≥ 2, demonstrating a positive predictive value of 62.5% and a negative predictive value of 94.4% in the derivation cohort. This revised score outperformed the original “rule of 5s” score (which used thresholds of 0.5 mm thickness, 5 mm length, and arc > 180°), particularly in patients with severely calcified lesions [21].

Intravascular imaging modalities, including intravascular ultrasound (IVUS) and optical coherence tomography (OCT), play a crucial role not only in characterizing the plaque, but also in guiding treatment decisions. These techniques allow for precise lesion assessment, and specific cut-off values have been established to identify hemodynamically significant stenoses and ensure optimal PCI outcomes. For IVUS, a minimum lumen area (MLA) < 4.0 mm^2^ in non-left main coronary arteries and <6.0 mm^2^ in the left main segment suggests a need for revascularization [25,26].

### 3.2. Left Main Coronary Artery Lesion Assessment and PCI Guidance

The use of intravascular ultrasound (IVUS) to guide PCI of the left main coronary artery (LMCA) is of paramount importance due to the unique anatomical and clinical challenges associated with this high-risk lesion subset. The LMCA supplies a vast myocardial territory, and suboptimal stent deployment can lead to catastrophic consequences, including stent thrombosis and restenosis with adverse clinical outcomes. For this reason, the latest ESC guidelines emphasize the pivotal role of intracoronary imaging in the assessment and optimization of LMCA interventions, granting a Class I recommendation for the use of intravascular imaging, particularly IVUS, to guide and evaluate stent implantation, ensuring adequate expansion and apposition and minimizing procedural complications [8]. IVUS provides detailed cross-sectional imaging, allowing for precise lesion assessment, plaque burden quantification, and optimal stent sizing. Several studies have demonstrated that IVUS-guided PCI of the LMCA leads to improved procedural and long-term outcomes. The MAIN-COMPARE study [27] and the ULTIMATE trial [28] showed that IVUS-guided PCI was associated with lower rates of major adverse cardiovascular events (MACEs) compared to angiography-guided PCI, primarily due to better stent expansion, apposition, and reduced edge dissections.

IVUS is crucial for the assessment of lesion severity, helping differentiate true significant stenoses from angiographically ambiguous lesions. The LITRO study defined with IVUS a minimal luminal area (MLA) of 6 mm^2^ as a cut-off to predict ischemia [29], showing poor outcomes at 2 years of patients with LM MLA < 6 mm^2^ who were not not revascularized. Subsequently Park, S.J. et al. identified a minimal luminal area (MLA) cut-off of 4.5 mm^2^ with IVUS corresponding to an FFR ≤ 0.80 [30]. Combining the results from these studies, the European Association of PCI currently suggests to revascularize LMCA with a MLA < 4.5 mm^2^, not to revascularize LM with a MLA > 6 mm^2^, and to consider functional assessment with a MLA between 4.5 and 6 mm^2^ [29].

As already mentioned, IVUS also provides information regarding plaque morphology and the need for lesion preparation in case of calcified plaques, which may require lesion preparation with atherectomy or lithotripsy before stenting. Proper stent diameter and length selection based on IVUS measurements and post-stenting optimization reduce the risk of malapposition, underexpansion, thrombosis, and restenosis.

Few data are available regarding OCT use for LM intervention, which is still not recommended for ostial disease evaluation or short left mains, because of flow turbulence and residual blood artifacts during image acquisition. Of note, the ongoing EMPERATRIZ study (NCT06272643) is assessing the concordance between IVUS- and OCT-derived minimum luminal area in angiographically intermediate (25–60%) left main lesions. The study includes patients undergoing PCI when IVUS-MLA is <6 mm^2^ and OCT shows no thin-cap fibroatheroma, and is expected to provide further insights into the comparative value of these imaging modalities in LM assessment.

### 3.3. Acute Coronary Syndrome

Intravascular imaging is extremely helpful in ACS patients with unclear culprit lesions. It can detect luminal irregularity or plaque rupture and consequent thrombus, the typical clues of an “unstable” atherosclerotic lesion. In particular, OCT is the most precise technique to detect intra-luminal thrombus [31], being able to distinguish between white and red thrombus due to their different optical attenuating properties [32].

Beyond thrombus detection, OCT enables a detailed differential diagnosis of culprit lesion morphology in acute coronary syndromes [32]. Plaque rupture appears as a fibrous cap discontinuity with a cavity communicating with the lumen, whereas plaque erosion shows an intact cap with adherent thrombus and no rupture. Coronary dissection is identified by a double lumen and intimal flap with intramural hematoma. OCT also distinguishes red thrombus, with high backscattering and strong signal attenuation, from white thrombus, which appears homogeneous and less attenuating. This morphological characterization provides key insights into the underlying pathophysiology of ACS and supports tailored therapeutic strategies.

However, it should be noted that in the acute coronary syndrome setting, differentiating plaque rupture from plaque erosion using OCT may be challenging due to the frequent presence of high intraluminal thrombus burden, which can obscure detailed visualization of the underlying plaque morphology [31].

For IVUS-based evaluation of culprit lesion plaque morphology and thrombotic burden, standardized definitions have been proposed [33]. Thrombus is identified as an intraluminal mass characterized by a layered, lobulated, or pedunculated appearance [34]. Overall, three thrombus subtypes can be distinguished by IVUS imaging [31]. Acute thrombus, reflecting platelet aggregation, appears as an acoustically inhomogeneous and bright intraluminal structure with sharp borders and without relevant signal attenuation. Subacute thrombus exhibits a more homogeneous echogenic pattern, resulting in a darker appearance. The most homogeneous form is organized thrombus, representing granulation tissue, which presents as a uniformly dark mass originating from the luminal surface, with well-defined margins and occasionally mild attenuation.

In addition to thrombus classification, IVUS analysis includes quantification of the maximum thrombus arc and the number of thrombus-containing frames, evaluated both at the culprit site and throughout the pre-intervention pullback. Plaque rupture is also assessed, defined as an intimal discontinuity with plaque ulceration and fibrous cap disruption, with or without intraluminal blood speckling within a cavity behind the ruptured cap [34,35].

Apart from identifying culprit atherosclerotic plaques, intracoronary imaging currently has a crucial role in the setting of myocardial infarctions with non-obstructed coronary arteries (MINOCAs), improving the diagnosis [36,37]. Many different etiologies coexist in this definition, including angiographically undetectable plaque disruption, coronary thromboembolism, coronary artery spasm, spontaneous coronary artery dissection (SCAD), myocarditis, and takotsubo syndrome [38]. In this heterogeneous scenario intravascular imaging plays a central role discerning between a plaque-induced event compared to a non-atherosclerotic etiology and hypothetically minimizing exposure to acute antiplatelet therapies.

Intravascular imaging has increased the awareness of SCAD in interventional community [39]. Despite the fact that a good amount of SCAD can be diagnosed by angiography alone, limiting the shift of catheters in a dissected vessel and the risks of dissection propagation and vessel closure, recent ESC/ACCA position papers suggest the use of intravascular imaging where angiographic diagnosis remains uncertain (commonly types 3 and 4 of SCAD classification) or in the event of hemodynamic instability [40]. Both IVUS and OCT can be used in a SCAD scenario, with pros and cons. The first concern about OCT regards contrast injection, which is a reason why IVUS is preferred when a false lumen is present (type 1 dissection).

### 3.4. Chronic Total Occlusion

Intravascular imaging can be crucial during chronic total occlusion (CTO) PCI, helping clarify the location of the proximal cap, particularly when located at the level of a bifurcation. Currently, IVUS represents the preferred intravascular imaging modality during CTO PCI, as it facilitates accurate guidewire positioning and cap penetration, thereby improving both procedural safety and efficiency. It also allows for confirmation of the wire location within the true distal lumen before stent implantation and supports re-entry maneuvers following subintimal tracking [41,42]. Beyond wire guidance, IVUS provides fundamental information on lesion length, plaque morphology, and vessel diameter, enabling optimal stent selection, expansion, and apposition. In this setting, IVUS is the imaging tool of choice. Notably, angiography alone frequently underestimates proximal and distal reference diameters compared with IVUS measurements, which may lead to inappropriate stent sizing; IVUS-guided optimization has been associated with favorable outcomes and low target lesion revascularization (TLR) rates at 6-month follow-up [43].

Compared with IVUS, OCT has been less frequently employed in CTO interventions. The lack of real-time imaging limits its usefulness for re-entry guidance in cases of proximal cap ambiguity or during complex reverse-CART techniques [44]. However, due to its superior resolution, OCT is an excellent modality for post-stenting assessment, particularly for detecting thrombus, tissue prolapse, edge dissections, and stent malapposition following successful CTO recanalization [22]. As a result, OCT is primarily used as a research tool to evaluate vascular healing after CTO PCI rather than in routine practice, where IVUS remains predominant. OCT studies have reported high rates of strut malapposition and delayed coverage after CTO procedures [45]. Furthermore, OCT use may be limited by the risk of dissection propagation during contrast injection, and image quality may be suboptimal in the absence of restored antegrade flow.

## 4. Intravascular Imaging Used to Define Vulnerable Plaque

It is known from studies based on histology and intracoronary imaging modalities that plaques prone to rupture and more likely to complicate are typically characterized by a large superficial lipid core covered by a thin fibrous cap and often an increased presence of inflammatory cells (about 26% infiltration of macrophages, mast cells, and neutrophils in the fibrous cap of vulnerable plaques compared with 3% in stable plaques) [46]. The size of a plaque or the severity of stenosis determined by it provides only part of the information about the vulnerability of the plaque [47]. Most thrombus-related acute coronary syndromes evolve from plaques determining only mild-to-moderate stenosis [48].

Intravascular imaging techniques have proven to be effective solutions for identifying high-risk plaques, shedding light on the role of extensive lipid accumulation, plaque burden, presence of local mediators of inflammation, and reduced lumen area in promoting acute coronary syndromes [49]. OCT in particular, with its ability to examine the surface components of plaque at high resolution, has the potential to identify high-risk plaques [50].

Definitions and diagnostic thresholds for OCT markers of plaque vulnerability were established on the basis of expert consensus documents and landmark intravascular imaging studies [16,51,52]. A coronary plaque is classified as high-risk when the following OCT features are identified: a minimum lumen area (MLA) < 3.5 mm^2^ across the analyzed segment, a fibrous cap thickness (FCT) < 75 µm at its thinnest point, a lipid-rich core with a lipid arc > 180°, and clusters of macrophages. Additional OCT findings may include intimal neovascularization, layered intraplaque tissue, cholesterol crystals, and calcified nodules—defined as nodular calcium deposits with overlying fibrous cap disruption. Notably, the CLIMA study demonstrated that the coexistence of four high-risk OCT characteristics—FCT < 75 µm, lipid arc > 180°, macrophage infiltration, and MLA < 3.5 mm^2^—correlates with a significantly increased incidence of major adverse cardiac events (MACE) at one-year follow-up [17].

The clinical relevance of OCT-detected thin-cap fibroatheroma (TCFA) in predicting adverse outcomes has been further demonstrated by the COMBINE OCT-FFR study [53]. In this prospective, multicenter trial including diabetic patients with FFR-negative lesions, the presence of at least one TCFA was associated with a five-fold higher rate of major adverse cardiovascular events (MACEs) at the 18-month follow-up compared to TCFA-negative lesions, despite the absence of ischemia by FFR. These findings highlight the limitations of a physiology-only approach and underscore the complementary role of high-resolution imaging in identifying morphologically vulnerable plaques that may confer substantial risk even in non-ischemic territories.

However, recent data have expanded the concept of high-risk plaque beyond the classical TCFA to include erosion-prone plaques and eruptive calcified nodules, which collectively represent the three major substrates of thrombosis in ACS. These evolving insights support a more comprehensive framework that integrates plaque morphology, composition, inflammatory activity, and hemodynamic factors [54].

Moreover, three-vessel OCT imaging studies from the Massachusetts General Hospital OCT Registry [55] showed that multiple high-risk plaques often cluster within the same patient and may be present even at non-culprit sites. By focusing on the treatment of morphologically high-risk but functionally non-significant plaques, the ongoing INTERCLIMA trial (NCT05027984) aims to determine whether a morphology-guided interventional approach can reduce the residual risk in patients with multivessel disease following ACS. It is a prospective, randomized clinical study designed to evaluate the clinical benefit of a preventive percutaneous coronary intervention (PCI) strategy targeting non-culprit coronary lesions characterized by high-risk features [56]. The rationale stems from the increasing recognition that vulnerable plaques—identified by intravascular imaging—can exist in angiographically non-significant segments and still lead to future adverse events. The results of this study may significantly influence future management paradigms, shifting from a physiology-based to a vulnerability-driven strategy for the treatment of coronary artery disease.

In addition, advances in artificial intelligence now allow for the simultaneous assessment of plaque morphology and computational physiology using optical coherence tomography (OCT). Through this approach, a novel morphologic metric—the lipid-to-cap ratio (LCR)—and a functional index derived from OCT to determine the hemodynamic relevance of coronary stenosis, known as the optical flow ratio (OFR), can be obtained alongside traditional morphologic parameters. The integration of morphologic and functional data appears to outperform either classical OCT-based vulnerability features or physiology-alone metrics in predicting future cardiovascular events [57].

## 5. The Role of Intravascular Imaging in Percutaneous Coronary Intervention: Stent Sizing, Optimization, Complication, and Clinical Outcomes

The use of intravascular imaging (IVI) for stent optimization has transformed contemporary PCI by providing precise vessel characterization, lesion assessment, and procedural guidance, ultimately reducing adverse clinical events. IVI, primarily IVUS and OCT, enhances stent implantation by improving lesion preparation, landing zone selection, stent sizing, and expansion assessment, while also aiding in the detection and management of acute procedural complications such as malapposition, edge dissection, and underexpansion [28].

Multiple randomized trials and meta-analyses have demonstrated that IVUS-guided PCI leads to superior procedural and long-term clinical outcomes compared to angiography-guided PCI [28,58,59]. IVUS-guided PCI results in more accurate vessel sizing, larger minimal stent area (MSA), and lower rates of stent thrombosis and target lesion failure (TLF). Early OCT trials, which initially used lumen-based sizing, resulted in smaller final MSA compared to IVUS [22]. However, subsequent studies, including ILUMIEN III and iSIGHT, demonstrated that an EEL-based sizing approach for OCT is non-inferior to IVUS guidance for final MSA and stent expansion [22,60].

While IVUS has consistently shown benefits in reducing major adverse cardiovascular events (MACEs), randomized data for OCT guidance remain limited. Real-world registries, however, have demonstrated that OCT-guided PCI is associated with lower in-hospital and long-term mortality compared to angiography guidance [61,62]. The ILUMIEN IV trial, currently ongoing, is expected to provide further clarity on whether OCT-guided PCI improves post-PCI luminal dimensions and clinical outcomes in patients with complex coronary disease or diabetes [63]. Additionally, the OCTOBER trial demonstrated that OCT-guided PCI in complex bifurcation lesions leads to lower rates of MACEs at 2 years compared to angiography-guided PCI [64]. The ROCK Cohort II study evaluated the safety and effectiveness of intravascular imaging—specifically optical coherence tomography (OCT)—as a diagnostic modality to guide left main angioplasty and assess corresponding mid-term outcomes. The investigators reported that intravascular imaging was superior to angiography for distal left main stenting, while showing no significant differences between OCT and IVUS [65].

### 5.1. Optimal Stent Landing Zones and Lesion Preparation

The ideal landing zones for stent implantation should be free of disease. In clinical practice, landing zones are identified distally and proximally to the lesion as segments as little diseased as possible (<50% plaque burden by IVUS [66] or visualization of >180° of EEL by OCT [32]). An excellent landing zone is represented by fibrous plaque [32], while lipidic plaques, particularly thin-cap fibroatheroma, should be avoided, to reduce the probability of an edge dissection and distal embolization [32]. Also, superficial calcification with an angle extending at 180° should be avoided as a landing segment. As a general rule, combined registration of intracoronary imaging and angiography, feasible with both IVUS and OCT, increases precision in localizing landing zones and reduces the risk of geographic miss [32].

Lesion preparation is particularly relevant in heavily calcified plaques, where adjunctive techniques such as rotational atherectomy, orbital atherectomy, or intravascular lithotripsy may be required for debulking before stent implantation to optimize expansion and reduce the risk of malapposition [67].

Appropriate reference vessel sizing is crucial for stent selection and postdilation to maximize stent expansion and lumen gain.

### 5.2. Stent Sizing and Optimal Expansion

IVI is extremely useful to choose stent diameter and length, because an incomplete lesion coverage represents a predictor of MACEs [68] and future stent failure, defined as stent restenosis or thrombosis [66,69,70]. As previously stated, deploying a stent on a landing zone characterized by residual plaque burden > 50% [66] and lipid-rich plaque increases the risk of stent edge restenosis and post-procedural MI [71], respectively. Therefore, intracoronary imaging should favor the choice of the best stent length by defining the most favorable landing zone. Co-registration of intracoronary imaging and angiography is an important tool to optimize the definition of the diseased segment and stent length and to simplify its deployment [72].

Angiographic assessment often underestimates both proximal and distal reference vessel dimensions, whereas IVUS reveals larger true vessel diameters, leading to the selection and implantation of significantly larger stent sizes [43].

Stent underexpansion, in fact, is a key predictor of early stent thrombosis and restenosis following drug-eluting stent (DES) implantation [73,74,75,76]. IVUS studies have demonstrated that pre-PCI assessment leads to larger stent diameter selection, greater minimal lumen diameter (MLD), and improved post-procedural stent expansion [77,78,79]. Moreover, incomplete lesion coverage has been associated with higher rates of MACEs and stent failure, including restenosis or thrombosis [80].

The post-PCI minimum stent area (MSA) represents the most powerful predictor of both restenosis and stent thrombosis [73,75,81,82,83,84,85]. Stent underexpansion, although frequently observed in both conditions, tends to be more pronounced and extensive in patients with stent thrombosis compared with those experiencing in-stent restenosis (ISR) [84]. IVUS data indicate that a post-procedural stent cross-sectional area of 5.5 mm^2^ is the optimal threshold for identifying lesions at higher risk of adverse outcomes in non-left main settings [81,86]. Similarly, in the DOCTORS trial, the best OCT-derived cut-off to predict a post-PCI fractional flow reserve (FFR) > 0.90 was an MSA > 5.44 mm^2^ [87]. In addition, Soeda et al. [85] demonstrated that an MSA < 5.0 mm^2^ independently predicted the 1-year device-oriented composite endpoint (DoCE)—including cardiac death, target vessel myocardial infarction, target lesion revascularization (TLR), and stent thrombosis—while Prati et al. found that an MSA < 4.5 mm^2^ was associated with major adverse cardiac events (MACEs) [62]. As expected, higher MSA cut-offs are required for left main lesions, typically >7 mm^2^ for distal LM and >8 mm^2^ for proximal LM when assessed by IVUS.

Optimal stent expansion targets include achieving an MSA > 100% of the distal reference lumen area or >80–90% of the averaged proximal and distal reference lumen areas. In long lesions, an MSA exceeding the distal reference (>100%) has been associated with very low 1-year event rates (approximately 1.5%) in IVUS-guided PCI [88]. Accordingly, the European Association of Percutaneous Cardiovascular Interventions (EAPCI) Expert Consensus supports using both absolute criteria (MSA > 5.5 mm^2^ by IVUS or >4.5 mm^2^ by OCT) and relative expansion criteria (MSA/reference lumen area > 80%) to define optimal stent expansion [72].

Table 2 provides an overview of the major clinical trials that have evaluated percutaneous coronary intervention (PCI) guidance using different imaging modalities, including optical coherence tomography (OCT), intravascular ultrasound (IVUS), and angiography, highlighting their impact on procedural optimization and clinical outcomes.

### 5.3. Stent Malapposition and Edge Dissections: Clinical Implications

Stent malapposition and underexpansion may coexist or may occur as distinct phenomena. Malapposition can be observed acutely, immediately after PCI, or it may arise later as a consequence of vascular mechanisms such as inflammation and positive (outward) vessel remodeling. In the absence of stent underexpansion, acute malapposition detected by either OCT or IVUS has not been associated with adverse early or long-term clinical outcomes, irrespective of its extent (length or thickness) [89,90,91,92].

**Figure 5:**
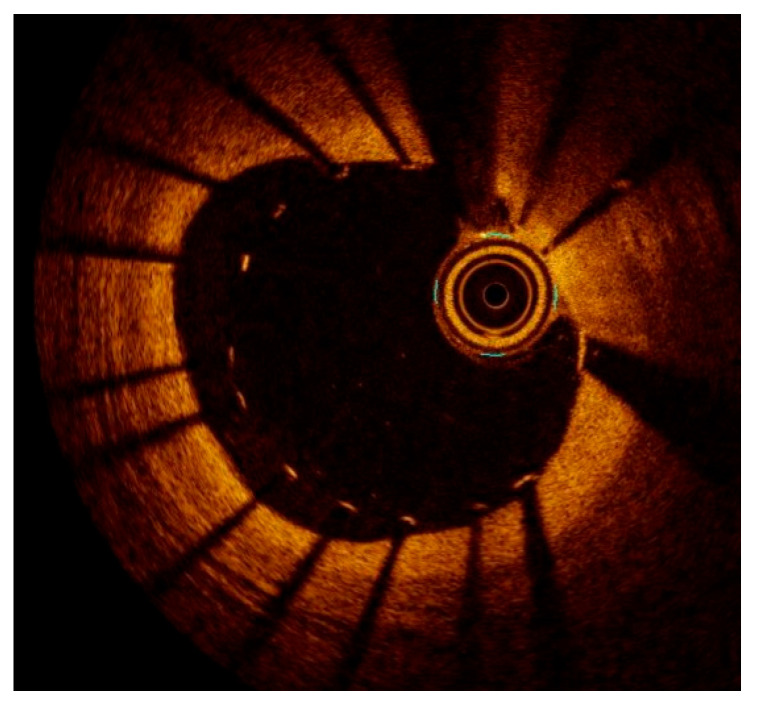
OCT imaging of stent malapposition. To note: malapposed stent struts, identifiable as bright structures with trailing shadows located at a distance from the intimal surface, without contact with the vessel wall.

The two intravascular imaging modalities also differ in their sensitivity for detecting malapposition, with OCT identifying it more than twice as frequently as IVUS [22,93] (Figure 5). Consistently, large cross-sectional IVUS and OCT studies have reported a prevalence of acute malapposition ranging from 8 to 15% with IVUS [92,94,95] and 39–62% with OCT, with approximately half of these cases resolving spontaneously at 9-month follow-up [62,85,90,91]. The long-term clinical implications of malapposition after DES implantation remain a matter of debate. While acute stent malapposition (ASM), regardless of its severity, has not shown a correlation with adverse outcomes across IVUS and OCT studies, accumulating evidence indicates that late-acquired stent malapposition (LASM) in first-generation DES may predispose to late or very late stent thrombosis (>1 year after PCI), likely driven by chronic inflammatory responses [96]. Conversely, the prognostic significance of late persistent stent malapposition (LPSM) is less clear, and this association appears to be attenuated with newer-generation DES featuring thinner struts and more biocompatible or biodegradable polymers [97].

Yonsei OCT Registry demonstrated that most suboptimal OCT findings were not associated with clinical outcomes, but a smaller minimal stent area was associated with device oriented clinical endpoint, driven mainly by target lesion revascularization, and significant malapposition with total malapposition volume (TMV) ≥ 7.0 mm^3^ was associated with more major safety events after PCI [98].

The latest consensus document from the European Association of Percutaneous Cardiovascular Interventions advises intervention in cases of acute stent malapposition (ASM) when the axial distance is ≥400 μm or the longitudinal extension is ≥1 mm [72]. This recommendation is supported by OCT studies demonstrating that ASM ≥ 400 μm tends to persist over time, as well as by three OCT-based stent thrombosis registries identifying malapposition as a major contributor to late and very late stent thrombosis [99,100].

**Figure 6:**
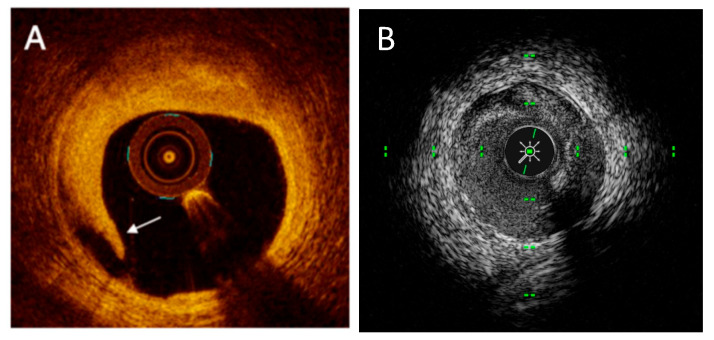
(**A**). OCT imaging of intravascular dissection. (**B**) IVUS imaging of intravascular dissection.

With regard to stent edge dissections (Figure 6), the superior resolution of OCT allows for the detection of small and less extensive dissections that are frequently missed by IVUS, as demonstrated in the ILUMIEN III trial [22]. However, OCT has been criticized for generating an excess of information, identifying edge dissections in up to 40% of PCI cases [22], approximately 80% of which are not visible on angiography. Importantly, the majority of OCT-detected dissections heal without clinical consequences at one-year follow-up [101].

Greater attention should instead be directed toward major edge dissections, as OCT-identified significant dissections have been shown to predict adverse outcomes [61,62,101,102]. Prati et al. reported that a distal rim of tissue >200 μm in width was associated with a 2.5-fold increased risk of major adverse cardiac events (death, myocardial infarction, or target lesion revascularization), whereas the same finding at the proximal edge did not carry the same prognostic weight [61,62]. Similarly, Van Zandvoort et al. [101] found that cavity depth at the distal edge, proximal reference lumen area, and total dissection length were independent predictors of the 1-year device-oriented composite endpoint. In line with these observations, the ILUMIEN IV stent optimization protocol recommends additional stent implantation in the presence of a major edge dissection, defined as ≥60° of vessel circumference involvement and ≥3 mm in length.

Furthermore, in the context of dissection (both spontaneous or iatrogenic) intravascular imaging, and IVUS in particular, may be used not only to assess the dissection but also the wire position (true lumen vs. subintimal) is unclear: the confirmation of the true-lumen guidewire position allows us to proceed with angioplasty, stent implantation, or other techniques. In this way it helps to avoid the risk of dissection propagation and subsequent abrupt vessel closure and to help the physician to re-enter the true lumen [103].

### 5.4. Intravascular Imaging in Stent Failure: In-Stent Restenosis and Stent Thrombosis

Intravascular imaging plays a crucial role in understanding the mechanism of stent failure: represented by in-stent restenosis (ISR) and stent thrombosis (ST).

Based on three-dimensional data obtained with intravascular imaging, in-stent restenosis (ISR) is characterized as a luminal re-narrowing of ≥75% of the reference vessel area on cross-sectional assessment [104]; this complication is frequent (reported in about 5% of all PCI procedures in Europe [105]) and a challenge to manage. Furthermore, a fundamental step in the ISR treatment algorithm is to define the cause of the restenosis [106] (Table 3), and intracoronary imaging has a key role in this diagnostic process.

The major cause of in-stent restenosis is stent underexpansion (in approximately 18–40% [73,107,108]), and other causes are stent fracture (<5%), neoatherosclerosis, and intimal hyperplasia. Current expert consensus documents and guidelines recommend the use of intravascular imaging (Class IIa, Level of Evidence B) for the assessment of ISR [72,109]. However, no randomized controlled trials to date have demonstrated that IVI-guided, morphology-based strategies should drive specific therapeutic approaches for ISR.

Stent thrombosis must be divided in early stent thrombosis (<30 days), mainly caused by malapposition, underexpansion edge dissections, late stent thrombosis (30–360 days), and very late stent thrombosis (>360 days), with the main causes being malapposition, neoatherosclerosis, uncovered struts, and underexpansion [72].

Both IVUS and OCT are able to define the cause of the stent failure; however, OCT provides superior diagnostic capability in the setting of late stent failure, as IVUS is unable to evaluate stent strut coverage or reliably identify thrombus, calcifications, or neoatherosclerosis (including lipid-rich, rupture-prone in-stent neointima) (Table 4). Notably, NIRS has also demonstrated the ability to detect lipidic neointimal tissue [110,111]. Conversely, IVUS should be more effective when the burden of thrombus reduces the resolution of the OCT.

According to OCT characteristics [112], ISR can be categorized into four distinct patterns:

- Homogeneous: a uniform, high-signal-intensity pattern with minimal backscatter, typically reflecting neointimal tissue rich in smooth muscle cells;
- Heterogeneous: a mixed-signal-intensity appearance, suggestive of proteoglycan-rich neointima or early neoatherosclerotic changes;
- Attenuated: a superficial, high-intensity signal with marked backscatter, most consistent with lipid-laden neoatherosclerotic plaque;
- Layered: a configuration most often characterized by a superficial high-intensity band overlying a deeper low-intensity layer, frequently localized around stent struts.

## 6. Novel Technologies

Since the first utilization of ultrasound technology in the coronary arteries, detecting plaques at risk of thrombotic events has been one of the main focuses of intravascular imaging.

Over the years, the phenotype of disrupted plaques has been identified, the thin-cap fibroatheroma (TCFA), the characteristics of which are large plaque burden [113], macrophage rich content covered by a thin fibrous cap [114], lipidic necrotic core, spotty calcification, and the presence of neovascularization [115].

Therefore, cardiovascular imaging in recent years turned his attention on the biochemical characterization of coronary atherosclerosis and morphologic features, aiming to estimate the biologic behavior of the plaque and predict future events.

Multimodal imaging with intravascular ultrasound (IVUS) or optical coherence tomography (OCT) has than the merit of joining biologic and anatomic characteristics of the plaques at risk.

### 6.1. NIRS

To improve the ability of IVUS determining plaque composition, fused NIRS-IVUS imaging has been suggested. Near-infrared spectroscopy imaging provides data about lipidic content in vessel walls, indicating the likelihood of the presence of lipid-rich plaques (LRPs). Quantification of the lipidic burden can be performed with the lipid-core burden index (LCBI): this index is obtained by dividing the number of yellow pixels by the total of pixels available and multiplying by 1000 (LCBI ranges from 0 to 1000). The LCBI 4 mm is used in clinical practice to quantify lipidic burden by dividing the interventional target region into 4 mm coronary segments [116]. Combined NIRS-IVUS can also reduce interindividual variability in the quantification of lipidic burden using the computerized LCBI [117,118]. The LRP study first demonstrated that the presence of lipid-rich plaque (defined by a LCBI 4 mm > 400) increased the event rate by four time [119].

Looking forward, there are four ongoing prospective trials that investigate LCBI or LCBI 4 mm as predictor of coronary events [120,121,122,123].

### 6.2. NIRF 

Near-infrared fluorescence (NIRF) uses near-infrared light to excite and detect fluorescent chemical compounds [124]. NIRF employs exogenous contrast agents, composed by fluorophores, used as tracers, conjugated to antibodies, peptides, or other biologically active molecules [125]. The value of NIRF resides in its wide range of applications thanks to the use of specifically targeted fluorophores.

From a practical perspective, multimodal NIRF-IVUS and NIRF-OCT catheters have already been designed [126,127].

The most used fluorophore is Indocyanine green (ICG), and it has been approved for clinical use since the 1970s. NIRF with ICG administration can detect plaque lipidic core because ICG specifically marks lipid-loaded macrophages [128].

MMPs and cathepsin are active proteases degrading atherosclerotic plaques and they have already been tested as alternative targets of NIRF agents [129,130]. Otherwise, ectopic calcification, one of the traits of the thin-cap fibroatheroma phenotype, can be detected by NIRF with bisphosphonate or hydroxyapatite-targeted fluorophore [131].

However, there are some remaining concerns before clinical application. To date, only ICG is approved for clinical use; the safety of other NIRF contrast agents in clinical application must therefore be investigated. And secondly, diagnostic accuracy of each fluorophore in human trials need to be tested.

### 6.3. NIRAF

NIRF imaging modality can even detect fluorescence from endogenous molecules, giving rise to the near-infrared autofluorescence (NIRAF) technique. The great advantage of NIRAF is the absence exogenous contrast agents. For that reason, NIRAF has already been approved in a first in-human study.

Ughi et al. [126] tested the safety and feasibility of the NIRAF-OCT system in 12 patients without device-related complications. NIRAF intensity areas matched to high-risk plaques detected by OCT. Multimodal NIRAF-OCT provided images identifying necrotic cores as safely as traditional imaging devices.

### 6.4. IVPA

Intravascular photoacoustic imaging (IVPA) is an emerging spectroscopic modality designed to characterize and differentiate vascular tissue components in vivo [132,133]. This modality sends short laser waves into the vessel wall and receives photoacoustic waves generated by thermal expansion of the tissue [134]. By analyzing receiving signals from the vessel wall, IVPA conceives plaque composition [133]. IVPA uses, as does NIRS, the differences in the optical absorption spectra of distinct tissues to detect plaque composition, but IVPA can provide deeper penetration and better resolution. IVPA can be used with endogenous agents or exogeneous contrast factors to detect specific molecular targets.

To date, IVPA clinical use remains distant because it still operates at low imaging speed and with relatively too large imaging probes to be tested in human trials.

### 6.5. FLIm

Fluorescence lifetime imaging (FLIm), like NIRAF, uses the autofluorescence properties of tissues to discern vessel wall composition. When irradiated with ultraviolet waves, the components of plaque (macrophages, LDL, or collagen) can be identified by detecting the lifetime of their reflected autofluorescence. Macrophages, foam cells, or collagen distribution on vessel wall affect autofluorescence lifetime and can identify plaques at risk [135,136]. FLIm does not need exogenous contrast agents but needs blood clearance due to the high ultraviolet light absorption of blood cells [136]. However, depth penetration of ultraviolet light is limited (around 200 μm) [137], which means that FLIm can provide information about the superficial arterial wall. 

An integrated FLIm-OCT catheter has already been designed and tested in vivo in animals [127]. A different FLIm-IVUS dual-modality imaging catheter has been validated using ex vivo human coronary arteries [135]. Building upon these successful pre-clinical trials, a first FLIm-OCT in vivo human study is planned in patients undergoing PCI [127].

### 6.6. Artificial Intelligence Integrated Software Analysis

Artificial intelligence (AI) is ushering in a new era of precision in intravascular imaging by enhancing image interpretation, procedural planning, and decision-making in PCI, and overcoming some of its limitations by automating key analytical tasks.

The AVVIGO+ IVUS system (Boston Scientific) incorporates a machine learning-based tool known as Automated Lesion Assessment (ALA), which uses U-Net convolutional neural networks to perform accurate lumen and vessel segmentation, achieving over 92% agreement with expert-determined balloon sizing [138]. It also automatically calculates plaque burden and identifies the MLA, allowing for precise stent sizing and landing zone selection while minimizing the risk of restenosis associated with high plaque burden areas.

The Ultreon 2.0 OCT platform (Abbott Vascular) leverages deep learning to detect and quantify calcific plaque (arc, thickness, length), which is essential for calcium scoring and planning pre-PCI plaque modification [23]. It also automates identification of the EEL, enabling precise reference measurements and enhancing device size selection and stent expansion. Clinical studies have shown that these AI-assisted features improve both the efficiency and accuracy of image interpretation, even among less-experienced operators [139].

The Gentuity HF-OCT system (Nipro), with its fast-pullback capability and low-profile catheter, provides high-resolution images while minimizing contrast use. It employs AI for real-time lumen segmentation, MLA localization, and detection of stent struts and expansion index. Notably, it performs reliably even under suboptimal conditions such as incomplete blood clearance or side branch interference, ensuring robust quantitative assessment throughout the imaging pullback [140].

The SpectraWAVE HyperVue system integrates DeepOCT and near-infrared spectroscopy (NIRS) in a single platform, enabling detailed tissue characterization. Its AI capabilities include automated detection of lipid-rich plaques and calcified lesions, quantitative plaque burden analysis, stent and MSA detection, and EEL delineation. These features support the identification of high-risk vulnerable plaques (e.g., thin-cap fibroatheromas), guiding pre-PCI planning and post-PCI optimization [141,142,143]. Moreover, automated pullback triggering and coregistration with angiography streamline the procedural workflow.

Despite these advancements, current AI-IVI systems face challenges, such as limitations in detecting qualitative features like stent edge dissections or thrombi, and vulnerability to image artifacts from calcifications or poor blood clearance. Furthermore, most systems are trained on datasets labeled by expert operators rather than histopathological gold standards, limiting generalizability. Nonetheless, future directions include integration with computational simulations, extended reality, and multimodal data (e.g., hemodynamic indices, genomics), paving the way for highly personalized PCI strategies [144].

In conclusion, AI-assisted IVI platforms significantly enhance the accuracy, reproducibility, and clinical utility of intravascular imaging. By automating complex analyses and supporting real-time decision-making, these technologies not only improve PCI precision but also represent a crucial step toward the broader adoption of imaging-guided, personalized coronary interventions.

## 7. Barriers and Facilitators to Clinical Adoption

Despite robust evidence supporting intravascular imaging-guided PCI, its widespread adoption in real-world practice remains limited [9]. Key barriers include additional procedural time, increased costs, and the need for specialized operator training to ensure accurate image acquisition and interpretation. Variability in device availability and the absence of standardized reimbursement policies further hinder its routine use.

Conversely, growing awareness of the prognostic benefits of imaging-guided interventions, integration into contemporary ESC [8] and AHA/ACC guidelines [7], and the emergence of artificial intelligence-assisted platforms are expected to facilitate broader implementation. Dedicated educational programs and workflow-optimized imaging protocols could also enhance efficiency and operator confidence.

Future research should focus on cost-effectiveness analyses, practical integration of AI-driven decision support systems, and real-world implementation strategies to bridge the gap between technological innovation and daily clinical practice.

In this context, the routine implementation of intravascular imaging within cath lab represents a crucial step toward improving procedural quality and patient outcomes. Standardized use of IVUS and OCT in complex PCI can significantly enhance lesion assessment, stent optimization, and complication management, reducing variability in operator-dependent decisions. Integrating imaging guidance into procedural protocols and training curricula is essential to ensure consistent interpretation and technical proficiency across centers. Furthermore, the incorporation of imaging data into digital cath lab ecosystems, combined with AI-driven analysis and real-time feedback, may streamline workflow and support evidence-based decision-making. The widespread clinical adoption of intravascular imaging should therefore be viewed not only as a technological advancement but as a fundamental component of precision interventional cardiology.

**Figure 7:**
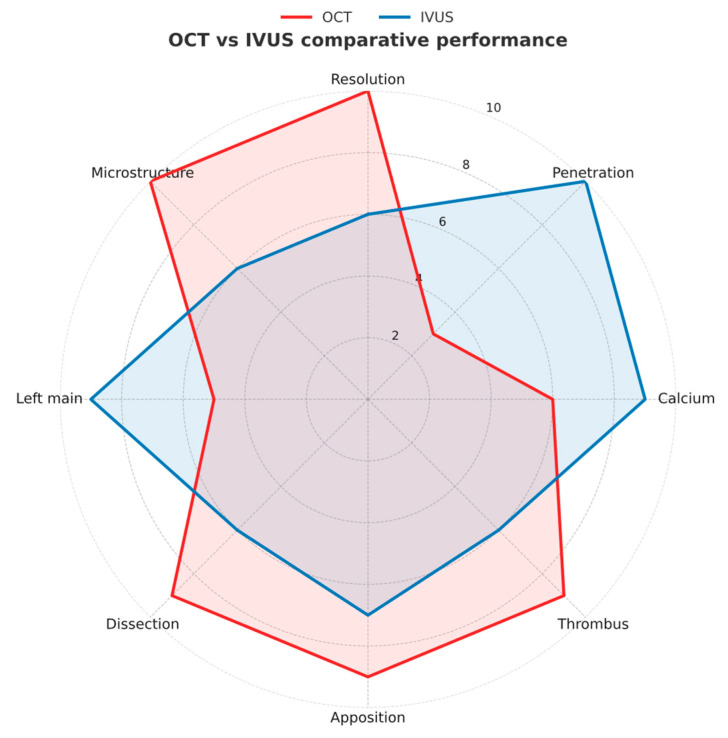
Comparative radar chart illustrating the relative performance of intravascular ultrasound (IVUS) and optical coherence tomography (OCT) across key procedural and diagnostic domains.

The radar chart in Figure 7 illustrates the comparative strengths of OCT and IVUS across key procedural and diagnostic domains. OCT provides superior axial resolution and excels in assessing stent apposition, thrombus burden, and microstructural plaque features, whereas IVUS offers deeper tissue penetration, superior calcium characterization, and greater accuracy for left main evaluation—features essential for complex and calcified lesions. This graphical comparison may help readers better appreciate the complementary roles and clinical applicability of both imaging modalities, emphasizing the importance of selecting the most appropriate technique based on lesion morphology and procedural objectives.

## 8. Conclusions

Over the past decades, intravascular imaging has significantly enhanced our understanding of coronary artery disease, offering high-resolution visualization of plaque morphology, lesion severity, and vascular response to PCI. IVUS and OCT have emerged as indispensable tools for guiding interventional procedures and identifying high-risk plaques that may predispose patients to adverse cardiovascular events. Their integration into routine clinical practice has led to improved patient outcomes, reducing complications such as stent thrombosis and restenosis.

Despite these advancements, challenges remain, including cost considerations, operator expertise, and the need for streamlined workflows to ensure broader adoption. Additionally, ongoing research aims to further refine imaging modalities, incorporating artificial intelligence and machine learning for automated plaque characterization, real-time image interpretation, and predictive analytics. Novel imaging technologies, such as NIRS and hybrid imaging approaches, hold promise for enhancing diagnostic precision and expanding clinical applications.

Looking ahead, the future of coronary intravascular imaging lies in the integration of multimodal techniques, improving accuracy while minimizing invasiveness. As technological innovations continue to evolve, their incorporation into clinical guidelines and real-world practice will be essential to maximize their impact on patient care. Ultimately, the goal remains to leverage these tools to advance precision medicine, enabling tailored therapeutic strategies and improved long-term cardiovascular outcomes.

## Figures and Tables

**Table 1:** Technical characteristics of IVUS and OCT catheters.

	IVUS (40–45 MHz)	HD IVUS (50–60 MHz)	OCT Frequency Domain
Wave source	Ultrasound	Ultrasound	Near-infrared light
Axial resolution (μm)	38–46	20–40	15–20
Penetration depth in soft tissue (mm)	>5	3–8	1–2
Distance between adjacent frames (mm)	0.02–0.03	0.02–0.17	0.1–0.25

**Table 2:** Clinical trials comparing OCT, IVUS, and/or angiography for PCI guidance. MACE = major adverse cardiac event, MSA = minimal stent area, FFR = fractional flow reserve, TVF = target vessel failure.

Study	Year of Publication	Study Design	Patients (n.)	ACS (%)	Stent Type	Follow-Up (Months)	Primary Endpoint
CLI-OPCI	2012	Retrospective (OCT vs. angiography)	670	60	DES or BMS	12	OCT-guidance PCI is associated with significantly lower rate of the composite of MACEs at 1 year
OCTACS	2015	Randomized (OCT vs. angiography)	100	100	DES	6	OCT-guided optimization improves stent strut coverage at 6-month follow-up
IVUS-XPL	2015	Randomized (IVUS vs. angiography)	1400	49	DES	12	IVUS-guided PCI is associated with significantly lower rate of the composite of MACEs at 1 year
ILUMIEN III	2016	Randomized (IVUS vs. OCT vs. angiography)	450	36	DES	1	OCT in MSA was non inferior vs. IVUS and not superior vs. angiography
DOCTORS	2016	Randomized (OCT vs. angiography)	240	100	DES vs. BMS	6	OCT-guidance PCI is associated with significantly greater post procedural FFR
OPINION	2017	Randomized (OCT vs. IVUS)	829	12	DES	12	TVF by OCT at 1 year—non-inferiority compared to IVUS guidance
Choi et al.	2019	Prospective registry (IVUS vs. angiography)	6005	37	DES	64	IVUS-guided PCI is associated with significantly lower risk of cardiac death
ROCK II	2021	Retrospective (OCT/IVUS vs. angiography)	730	60	DES	12	Intravascular imaging was superior to angiography for distal LM stenting, with no difference between OCT and IVUS.
OCTOBER	2023	Randomized (OCT vs. angiography)	1201	45	DES	24	OCT-guided PCI was associated with a lower incidence of MACEs in patients with complex coronary-artery bifurcation lesions

**Table 3:** Clinical, anatomical, and procedural factors associated with in-stent restenosis (ISR). CKD: chronic kidney disease; CV: cardiovascular; CTO: chronic total occlusion; SVG: saphenous vein graft; PCI: percutaneous coronary intervention; MLA: minimal lumen area.

Clinical Factors	Anatomical Factors	Stent/Procedural Factors
Chronic kidney disease (CKD)	Calcified lesion	Bare metal stent
Diabetes mellitus	Ostial lesion	Stent underexpansion
Older age	Bifurcation lesion	Multiple stent layers
Obesity	Long lesion	Stent fracture
Antiproliferative drug resistance	Small vessel	Geographical miss
Untreated cardiovascular risk factors	Previous ISR	Stent gap
	CTO revascularization	Small post-PCI MLA
	SVG revascularization	Smaller stent diameter
		Longer stent

**Table 4:** Multimodality coronary imaging characterization of in-stent restenosis (ISR). The table summarizes the main morphological features of ISR as assessed by the two leading intracoronary imaging modalities: intravascular ultrasound (IVUS) and optical coherence tomography (OCT).

IVUS	
Focal ISR	Defined as a lumen area < 4.0 mm^2^ and a lesion length < 10 mm, further classified according to its location within the stented segment: Focal-body type: confined to the central portion of the stent Focal-marginal type: extending to the proximal or distal stent edges
Multifocal ISR	Characterized by multiple discrete restenotic segments, with the following subtypes: Multifocal-body type: limited to the stent body Multifocal-marginal type: involving one or both stent margins
Diffuse ISR	Defined as a minimum lumen area < 4.0 mm^2^ with a lesion length > 10 mm, and categorized as follows: Diffuse-body type: restricted to the stented shaft Diffuse-marginal type: associated with edge involvement
**OCT**	
Type I	Thin-cap neoatheroma: presence of one or more thin fibrous caps with neoatherosclerotic tissue located between the lumen and the stent struts
Type II	Thick-cap neoatheroma: neoatherosclerosis situated between the lumen and the stent struts without thin-cap features
Type III	Peri-strut neoatheroma: neoatherosclerotic tissue predominantly distributed around the stent struts
Type IV	Pre-existing fibroatheroma: native atherosclerotic plaque appearing as a signal-poor region with blurred borders between the stent struts and the adventitia

## Data Availability

No new data were created or analyzed in this study. Data sharing is not applicable to this article.

## Abbreviations

The following abbreviations are used in this manuscript:

ACS	Acute Coronary Syndrome
ADE	Automated Differential Echogenicity
AHA	American Heart Association
ALA	Automated Lesion Assessment
AI	Artificial Intelligence
BMS	Bare-Metal Stent
CCS	Chronic Coronary Syndrome
CKD	Chronic Kidney Disease
CART	Controlled Antegrade and Retrograde subintimal Tracking; including “reverse-CART”
CTO	Chronic Total Occlusion
DES	Drug-Eluting Stent
DoCE	Device-Oriented Composite Endpoint
EAPCI	European Association of Percutaneous Cardiovascular Interventions
EEL	External Elastic Lamina
ESC	European Society of Cardiology
FCT	Fibrous Cap Thickness
FFR	Fractional Flow Reserve
FLIm	Fluorescence Lifetime Imaging
HD-IVUS	High-Definition IVUS
IB-IVUS	Integrated Backscatter IVUS
IEL	Internal Elastic Lamina
iMAP-IVUS	Intravascular Ultrasound with Integrated Mapping Analysis of Plaque
ISR	In-Stent Restenosis
IVI	Intravascular Imaging
IVL	Intravascular Lithotripsy
IVPA	Intravascular Photoacoustic
IVUS	Intravascular Ultrasound
LCBI	Lipid Core Burden Index
LCR	Lipid-to-Cap Ratio
LM	Left Main
LMCA	Left Main Coronary Artery
LPSM	Late Persistent Stent Malapposition
LASM	Late Acquired Stent Malapposition
LRP	Lipid-Rich Plaque
MACE	Major Adverse Cardiac Events
MI	Myocardial Infarction
MLA	Minimum Lumen Area
MLD	Minimum Lumen Diameter
MSA	Minimum Stent Area
MINOCA	Myocardial Infarction with Non-Obstructive Coronary Arteries
NIRAF	Near-Infrared Autofluorescence
NIRF	Near-Infrared Fluorescence
NIRS	Near-Infrared Spectroscopy
OCT	Optical Coherence Tomography
OFDI	Optical Frequency Domain Imaging
OFR	Optical Flow Ratio
PCI	Percutaneous Coronary Intervention
RF	Radio Frequency
SCAD	Spontaneous Coronary Artery Dissection
ST	Stent Thrombosis
SVG	Saphenous Vein Graft
TCFA	Thin-Cap Fibroatheroma
TLF	Target Lesion Failure
TLR	Target Lesion Revascularization
TMV	Total Malapposition Volume
TVF	Target Vessel Failure
VH-IVUS	Virtual Histology IVUS
